# Supplementary material for: Conserved sequence motifs in the abiotic stress response protein late embryogenesis abundant 3
Source: PLoS One. 2020 Aug 6;15(8):e0237177. doi: 10.1371/journal.pone.0237177 (PMC7410210; doi:10.1371/journal.pone.0237177)
Supplement: S1 Fig — A plant species tree was generated using PhyloT and NCBI genomic reference numbers as described in the Materials and Methods. The five architectures for each LEA3 protein are listed. The total for each species listed in the right-hand column, and the total of each architecture are listed in the bottom row. (PDF) [file pone.0237177.s001.pdf]

Fig. S1

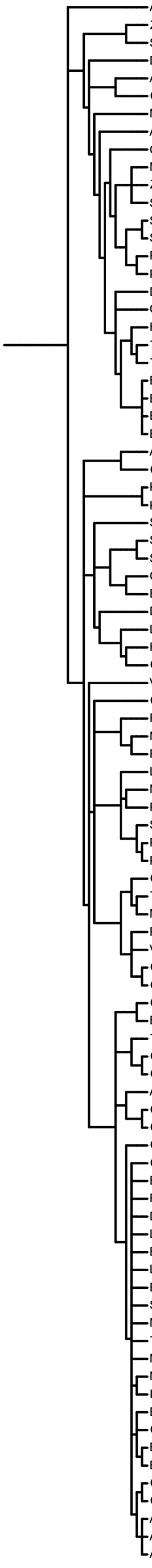

Species	MAARS 1-W	MAARS 2-W	MGRX 1-W	MASRKVI 1-W	MAARS 1-W **NO** DEALR	Total
Aquilegia coerulea	1		1	1		3
Zostera marina						0
Spirodela polyrhiza	1					1
Dioscorea alata	1					1
Asparagus officinalis	2					3
Cattleya violacea	1		1		1	2
Musa acuminata	1		1			2
Ananas comosus	2					2
Oropetium thomaeum	1					1
Miscanthus sinensis			2	6		8
Zea mays	1		2	1		4
Sorghum bicolor	3		1	2		6
Setaria italica				3	1	4
Setaria viridis	3		1	5		9
Panicum hallii	3		2	10		15
Panicum virgatum			2	5		7
Dendrocalamus strictus	3	1		1		5
Oryza sativa	1		1	4		6
Hordeum vulgare	1			1		2
Triticum aestivum				3		3
Thinopyrum intermedium	4		2	6		12
Brachypodium distachyon			1	3		4
Brachypodium hybridum	1		1	3		5
Brachypodium sylvaticum	1		1	4		6
Brachypodium stacei	1		1	3		6
Amaranthus hypochondriacus	3		2		2	7
Chenopodium quinoa	2					2
Kalanchoe fedtschenkoi	4			2		6
Kalanchoe laxiflora	12		2			14
Sarracenia purpurea	3		1			4
Solanum lycopersicum	3		1			4
Solanum tuberosum	2					2
Olea europaea	7					7
Erythranthe guttata	1					1
Daucus carota	2					2
Lactuca sativa	2					2
Helianthus annuus	2		4			6
Calendula incana subsp. maritima	7		2			9
Vitis vinifera	1		3			4
Cucumis sativus	2					2
Fragaria vesca	3		1			4
Malus domestica	1		2			3
Prunus persica	1		1			2
Linum usitatissimum	5					5
Manihot esculenta	4		2			6
Ricinus communis	1		1			2
Salix alba	8	1	5		1	15
Populus deltoides	2		2			4
Populus trichocarpa	3		1			4
Cicer arietinum	3		1			4
Trifolium pratense	2					2
Medicago truncatula	4		2			6
Phaseolus vulgaris	5		1			6
Vigna unguiculata	5		1			6
Glycine soja	7		2			9
Glycine max			1			1
Corymbia citriodora	3		1			4
Eucalyptus grandis	4	1				5
Theobroma cacao	2		1			3
Gossypium hirsutum	6		2			8
Gossypium raimondii	4		2			6
Anacardium occidentale	3		2			5
Citrus sinensis	3					3
Citrus clementina	2					2
Carica papaya	1		1			2
Caulanthus amplexicaulis	4		1		1	6
Eutrema salsugineum	3	1	1		1	6
Rorippa islandica	3		1			4
Descurainia sophioides	3	1	1		1	6
Lunaria annua	4	1	2			7
Boechera stricta	3	1	1			5
Lepidium sativum	5		2			7
Euclidium syriacum	3		2			5
Stanleya pinnata	5		1			6
Meniocus linifolius	4	2	2		1	9
Thlaspi arvense	3		1		1	5
Malcolmia maritima	3	1	1		1	6
Myagrum perfoliatum	3	1	1		1	6
Isatis tinctoria	3	5	2			10
Eruca vesicaria	13	1	5			19
Crambe hispanica	5	1	3			9
Brassica oleracea	3		2			5
Brassica rapa	6	1	3			10
Capsella rubella	3	1	1			5
Capsella grandiflora	2	1	1			4
Arabidopsis thaliana	3	1	1			5
Arabidopsis lyrata	3	1				4
Arabidopsis halleri	1	1	1			3
Total	256	24	103	63	12	458

Figure S1. LEA3 Proteins in Higher plants by species. A plant species tree was generated using PhyloT and NCBI genomic reference numbers as described in the Materials and Methods. The five architectures for each LEA3 protein are listed. The total for each species listed in the right-hand column, and the total of each architecture are listed in the bottom row.
