# Supplementary material for: Conserved sequence motifs in the abiotic stress response protein late embryogenesis abundant 3
Source: PLoS One. 2020 Aug 6;15(8):e0237177. doi: 10.1371/journal.pone.0237177 (PMC7410210; doi:10.1371/journal.pone.0237177)
Supplement: S1 Table — Plant species names are divided into higher plants, lower plants, and algae. (DOC) [file pone.0237177.s002.doc]

**S1 Table. List of species used in this study.** Plant species names are divided into higher plants, lower plants, and algae.

| **Higher Plants** | **Lower Plants** | **Algae** |
| --- | --- | --- |
| *Alyssum linifolium* | *Amborella trichopoda* | *Bathycoccus prasinos* |
| *Amaranthus hypochondriacus* | *Azolla filiculoides* | *Chlorella sp. NC64A* |
| *Amborella trichopoda* | *Ginkgo biloba* | *Chlamydomonas reinhardtii* |
| *Anacardium occidentale* | *Marchantia polymorpha* | *Coccomyxa subellipsoidea* |
| *Ananas comosus* | *Physcomitrella patens* | *Cyanidioschyzon merolae* |
| *Aquilegia coerulea* | *Picea abies* | *Dunaliella salina* |
| *Arabidopsis halleri* | *Salvinia cucullate* | *Fragilariopsis cylindrus* |
| *Arabidopsis lyrata* | *Selaginella moellendorffi* | *Micromonas pusilla* |
| *Arabidopsis thaliana* | *Sphagnum fallax* | *Micromonas sp. RCC299* |
| *Asparagus officinalis* |  | *Ostreococcus lucimarinus* |
| *Boechera stricta* |  | *Ostreococcus tauri* |
| *Brachypodium distachyon* |  | *Phaeodactylum tricornutum* |
| *Brachypodium hybridum* |  | *Thalassiosira pseudonana* |
| *Brachypodium stacei* |  | *Volvox carteri* |
| *Brachypodium sylvaticum* |  |  |
| *Brassica oleracea* |  |  |
| *Brassica rapa* |  |  |
| *Calendula maritima* |  |  |
| *Capsella grandiflora* |  |  |
| *Capsella rubella* |  |  |
| *Carica papaya* |  |  |
| *Cattleya violacea* |  |  |
| *Caulanthus amplexicaulis* |  |  |
| *Chenopodium quinoa* |  |  |
| *Cicer arietinum* |  |  |
| *Citrus clementina* |  |  |
| *Citrus sinensis* |  |  |
| *Corymbia citriodora* |  |  |
| *Crambe hispanica* |  |  |
| *Cucumis sativus* |  |  |
| *Daucus carota* |  |  |
| *Dendrocalamus strictus* |  |  |
| *Descurainia sophiodes* |  |  |
| *Dioscorea alata* |  |  |
| *Eruca vesicaria* |  |  |
| *Eucalyptus grandis* |  |  |
| *Euclidium syriacum* |  |  |
| *Eutrema salsugineum* |  |  |
| *Fragaria vesca* |  |  |
| *Glycine max* |  |  |
| *Glycine soja* |  |  |
| *Gossypium hirsutum* |  |  |
| *Gossypium raimondii* |  |  |
| *Helianthus annuus* |  |  |
| *Hordeum vulgare* |  |  |
| *Isatis tinctoria* |  |  |
| *Kalanchoe fedtschenkoi* |  |  |
| *Kalanchoe laxiflora* |  |  |
| *Lactuca sativa* |  |  |
| *Lepidium sativum* |  |  |
| *Linum usitatissimum* |  |  |
| *Lunaria annua* |  |  |
| *Malcolmia maritima* |  |  |
| *Malus domestica* |  |  |
| *Manihot esculenta* |  |  |
| *Marchantia polymorpha* |  |  |
| *Medicago truncatula* |  |  |
| *Mimulus guttatus* |  |  |
| *Miscanthus sinensis* |  |  |
| *Musa acuminata* |  |  |
| *Myagrum perfoliatum* |  |  |
| *Olea europaea* |  |  |
| *Oropetium thomaeum* |  |  |
| *Oryza sativa* |  |  |
| *Panicum hallii* |  |  |
| *Panicum virgatum* |  |  |
| *Phaseolus vulgaris* |  |  |
| *Physcomitrella patens* |  |  |
| *Populus deltoides* |  |  |
| *Populus trichocarpa* |  |  |
| *Prunus persica* |  |  |
| *Ricinus communis* |  |  |
| *Rorippa islandica* |  |  |
| *Salix alba* |  |  |
| *Sarracenia purpurea* |  |  |
| *Selaginella moellendorffi* |  |  |
| *Setaria italica* |  |  |
| *Setaria viridis* |  |  |
| *Solanum lycopersicum* |  |  |
| *Solanum tuberosum* |  |  |
| *Sorghum bicolor* |  |  |
| *Sphagnum fallax* |  |  |
| *Spirodela polyrhiza* |  |  |
| *Stanleya pinnata* |  |  |
| *Theobroma cacao* |  |  |
| *Thinopyrum intermedium* |  |  |
| *Thlaspi arvense* |  |  |
| *Trifolium pratense* |  |  |
| *Triticum aestivum* |  |  |
| *Vigna unguiculata* |  |  |
| *Vitis vinifera* |  |  |
| *Zea mays* |  |  |
| *Zostera marina* |  |  |
|  |  |  |
|  |  |  |
|  |  |  |
