# Supplementary material for: Conserved sequence motifs in the abiotic stress response protein late embryogenesis abundant 3
Source: PLoS One. 2020 Aug 6;15(8):e0237177. doi: 10.1371/journal.pone.0237177 (PMC7410210; doi:10.1371/journal.pone.0237177)
Supplement: Attachment — Submitted filename: Response to reviewers.doc [file pone.0237177.s004.doc]

Department of Molecular and Cellular Biology

Guelph, Ontario, Canada, N1G 2W1

Monday, November 30, 2026

RE: Response to reviewers for manuscript PONE-D-20-14558

Dear Editor,

Thank-you for the opportunity to submit a revision of our manuscript titled “Conserved Sequence Motifs in the Abiotic Stress Response Protein Late Embryogenesis Abundant 3.” We hope that addressing these issues will allow our manuscript to be published.

Response to the Editor

1) We have revised our manuscript to ensure that it follows the PLOS One formatting guidelines.
2) We have removed the “data not shown” statements and rewritten the text to indicate that a) no algae sequences were found so that there is no data shown and b) the data for the lower plant N-terminal sequences are shown in the supplemental data.

Response to Reviewer #1

1) We thank the reviewer for pointing out that our figures did not take color vision deficiency into account. We have corrected the Figures 1, 2, and 5 to ensure that the colors and color shades are appropriate now.
2) The suggestion of using PCA to see if there is any clustering of sequences by species is interesting, but one that we feel that the reviewer would not see as necessary if they were able to open Fig. S1. There does not seem to be any significant trend, other than our previously described observation that the M[AS][RK] N-terminal sequence is predominantly found in Commelinids. We are also unclear on what variables or categories would be used for the PCA. We therefore felt that adding a PCA analysis would not significantly improve the manuscript.
3) We corrected the upper MW limit of dehydrins (now Line 566 in the revised with track-changes manuscript).
4) We corrected ‘containing’ to ‘contain’ (now line 18) and ‘arose’ to ‘arisen’ (now line 28).

Sincerely,

Steffen Graether, Ph.D.

Associate Professor

Department of Molecular and Cellular Biology

University of Guelph

Guelph, Ontario

Email: graether@uoguelph.ca

Phone: 519-824-4120 ext. 56457
